# Estimated Number of Deaths Prevented Through Increased Physical Activity Among US Adults

**DOI:** 10.1001/jamainternmed.2021.7755

**Published:** 2022-01-24

**Authors:** Pedro F. Saint-Maurice, Barry I. Graubard, Richard P. Troiano, David Berrigan, Deborah A. Galuska, Janet E. Fulton, Charles E. Matthews

**Affiliations:** 1 Division of Cancer Epidemiology and Genetics, National Cancer Institute, Rockville, Maryland; 2 Division of Cancer Control and Population Sciences, National Cancer Institute, Rockville, Maryland; 3 Division of Nutrition, Physical Activity, and Obesity, National Center for Chronic Disease Prevention and Health Promotion, Centers for Disease Control and Prevention, Atlanta, Georgia

## Abstract

This cohort study uses National Health and Nutrition Examination Survey data to estimate the number of deaths that could be prevented through increased physical activity among US adults.

Previous studies suggest that a substantial number of deaths could be prevented annually by increasing population levels of physical activity.^1,2,3^ However, previous estimates have relied on convenience samples,^2,3^ used self-reported physical activity data,^1,2,3^ and assumed relatively large increases in activity levels (eg, more than 30 minutes per day).^1,2,3^ The potential public health benefit of changing daily physical activity by a manageable amount is not yet known. In this study, we used accelerometer measurements (1) to examine the association of physical activity and mortality in a population-based sample of US adults and (2) to estimate the number of deaths prevented annually with modest increases in moderate-to-vigorous physical activity intensity (MVPA).

## Methods

This cohort study was approved by the National Center for Health Statistics Ethics Review Board. This study used data from the National Health and Nutrition Examination Survey (NHANES), and written informed consent was obtained for all NHANES participants. The study followed the Strengthening the Reporting of Observational Studies in Epidemiology (STROBE) reporting guideline.

The NHANES is a representative survey of the US civilian, noninstitutionalized population, including oversampling for non-Hispanic Black participants and Mexican American participants. Race and ethnicity was determined by self-report and classified using preferred terminology from the National Center for Health Statistics as Mexican American, non-Hispanic Black, non-Hispanic White, or other. Race and ethnicity was included in this study to better characterize the US population. In 2003 to 2006, NHANES participants aged 6 years or older were asked to wear an accelerometer for 7 days. For this study, we evaluated 4840 of 6355 adults aged 40 to 85 years or older with accelerometer data. The remaining 1515 individuals were excluded because they were not eligible or refused to participate in the monitoring protocol (853 [13%]), had monitors that malfunctioned or lost calibration (360 [6%]), or had no valid days with monitor data (302 [5%]). Mortality follow-up was completed via National Death Index linkage through December 31, 2015. We estimated MVPA by summing accelerometer minutes at or above an established cutpoint^4^ and creating 8 physical activity categories (0-19, 20-39, 40-59, 60-79, 80-99, 100-119, 120-139, or ≥140 minutes per day).

The number of deaths per year prevented with increased physical activity was estimated as the adjusted population attributable fraction (PAF)^5^ multiplied by the US population annual number of deaths for 2003 (for individuals aged 40-84 years). To calculate the PAFs, we used population prevalence estimates and hazard ratios adjusted for age, sex, race and ethnicity, education level, body mass index (calculated as weight in kilograms divided by height in meters squared), diet, alcohol use, smoking status, and self-reported chronic conditions, mobility limitations, and general health. Hazard ratios were estimated using Cox proportional hazard regression models, and the proportional hazards assumption was confirmed for our main exposure (ie, MVPA). Counterfactuals for increased activity were set to 10, 20, and 30 minutes per day higher than participants’ observed values. Those classified as frail^6^ or who required equipment to walk were assumed to be unable to increase their activity (eMethods in the Supplement); when PAFs were calculated, physical activity levels for these participants were held constant. Data were analyzed with SAS version 9.4 (SAS Institute Inc), accounting for the NHANES complex sample design.

## Results

This analysis included 4840 participants. Of these, 2435 (53%) were women, 993 (10.4%) were non-Hispanic Black, and 887 (5.1%) were Mexican American (Table). A total of 1165 deaths occurred during a mean (SEM) follow-up of 10.1 (0.1) years.

**Table.** Characteristics of the US Population Aged 40 to 85 Years or Older by the Amount of MVPA, 2003 to 2006 NHANES^a^

Characteristic	MVPA, min/d^b^					*P* value^c^
	0-39.9 (n = 1164)	40.0-79.9 (n = 1249)	80.0-119.9 (n = 1141)	≥120.0 (n = 1286)	Total (N = 4840)	
Age, mean (95% CI), y	70.4 (69.3-71.6)	58.4 (57.5-59.3)	53.8 (53.0-54.7)	50.7 (50.1-51.3)	57.0 (56.3-57.6)	<.001
BMI, mean (95% CI)	29.3 (28.9-29.7)	29.9 (29.4-30.4)	29.0 (28.6-29.4)	27.8 (27.5-28.1)	28.9 (28.7-29.1)	<.001
Healthy Eating Index score, mean (95% CI)^d^	58.2 (57.3-59.1)	57.2 (56.4-58.0)	57.1 (56.1-58.0)	55.3 (54.3-56.3)	56.8 (56.2-57.3)	.01
MVPA, mean (95% CI), min/d	21.7 (21.1-22.3)	60.5 (59.9-61.1)	99.0 (98.1-99.8)	171.4 (168.9-173.9)	98.2 (95.6-100.7)	<.001
Sex						
Men	489 (34.7)	540 (39.2)	555 (44.4)	821 (62.4)	2405 (47.0)	<.001
Women	675 (65.3)	709 (60.8)	586 (55.6)	465 (37.6)	2435 (53.0)	
Race and ethnicity^e^						
Mexican American	149 (3.1)	213 (4.1)	208 (5.3)	317 (7.0)	887 (5.1)	.41
Non-Hispanic						
Black	213 (10.9)	283 (11.2)	240 (9.7)	257 (10.0)	993 (10.4)	
White	754 (81.2)	680 (76.7)	614 (77.4)	633 (75.8)	2681 (77.4)	
Other groups	48 (4.7)	73 (8.0)	79 (7.6)	79 (7.2)	279 (7.0)	
Education						
Less than high school	452 (30.4)	344 (16.2)	284 (13.6)	372 (14.8)	1452 (17.6)	.01
High school	300 (28.3)	331 (28.4)	265 (24.3)	291 (25.7)	1187 (26.5)	
More than high school	412 (41.3)	574 (55.5)	592 (62.1)	623 (59.5)	2201 (55.9)	
BMI^f^						
Normal weight	349 (28.4)	292 (24.2)	307 (28.3)	365 (30.8)	1313 (28.1)	<.001
Overweight	387 (32.9)	436 (33.9)	432 (36.5)	540 (39.2)	1795 (36.1)	
Obesity	428 (38.7)	521 (41.9)	402 (35.2)	381 (30.0)	1732 (35.8)	
Alcohol use						
Never	222 (18.1)	176 (13.1)	129 (8.9)	99 (6.6)	626 (10.8)	<.001
Former	248 (23.1)	244 (18.9)	192 (16.0)	171 (12.5)	855 (16.9)	
Current	628 (53.4)	759 (62.6)	753 (69.4)	937 (75.7)	3077 (66.8)	
Missing or unknown	66 (5.5)	70 (5.4)	67 (5.7)	79 (5.2)	282 (5.4)	
Smoking status						
Never smoker	506 (42.3)	582 (45.8)	567 (52.3)	579 (45.4)	2234 (46.8)	.46
Former smoker	473 (39.7)	406 (30.7)	344 (27.8)	344 (32.7)	1633 (32.2)	
Current smoker	185 (18.0)	261 (23.5)	230 (19.9)	230 (21.9)	973 (21.0)	
Comorbid conditions						
Diabetes mellitus						
Yes	310 (24.6)	199 (12.6)	127 (9.2)	89 (4.1)	725 (11.2)	<.001
No	820 (72.2)	1016 (85.1)	1001 (89.7)	1182 (94.9)	4019 (87.1)	
Borderline	34 (3.2)	34 (2.3)	13 (1.2)	15 (1.0)	96 (1.7)	
Stroke						
Yes	144 (12.1)	51 (3.2)	42 (2.7)	19 (1.0)	256 (4.0)	<.001
No	1020 (87.9)	1198 (96.8)	1099 (97.3)	1267 (99.0)	4584 (96.0)	
Coronary heart disease						
Yes	149 (13.1)	85 (6.0)	55 (3.7)	30 (2.2)	319 (5.5)	<.001
No	1001 (85.9)	1159 (93.8)	1080 (96.0)	1254 (97.7)	4494 (94.2)	
Missing or unknown	14 (0.9)	5 (0.2)	6 (0.3)	2 (0.2)	27 (0.3)	
Heart failure						
Yes	146 (12.8)	52 (3.1)	25 (1.6)	21 (1.3)	244 (3.9)	<.001
No	1002 (86.3)	1193 (96.8)	1115 (98.4)	1264 (98.6)	4574 (95.9)	
Missing or unknown	16 (0.9)	4 (0.1)	1 (0.0)	1 (0.1)	22 (0.2)	
Cancer						
Yes	266 (24.7)	167 (13.5)	128 (11.2)	79 (7.5)	640 (13.0)	<.001
No	898 (75.3)	1082 (86.5)	1013 (88.8)	1207 (92.5)	4200 (87.0)	
Chronic bronchitis						
Current	67 (6.7)	56 (5.5)	22 (2.2)	30 (2.4)	175 (3.9)	<.001
Former	60 (6.3)	53 (4.6)	33 (3.0)	34 (3.5)	180 (4.1)	
Never	1031 (86.3)	1138 (89.9)	1081 (94.4)	1218 (93.8)	4468 (91.7)	
Missing or unknown	6 (0.6)	2 (0.1)	5 (0.4)	4 (0.3)	17 (0.3)	
Emphysema						
Yes	86 (8.0)	40 (3.0)	17 (1.2)	12 (0.7)	155 (2.7)	<.001
No	1069 (91.3)	1209 (97.0)	1123 (98.7)	1272 (99.2)	4673 (97.1)	
Missing or unknown	9 (0.7)	0	1 (0.1)	2 (0.1)	12 (0.2)	
Mobility limitations^g^						
None	416 (34.8)	570 (37.3)	456 (32.0)	391 (22.7)	1833 (30.9)	<.001
Unrelated to mobility	42 (5.8)	323 (37.7)	511 (55.1)	769 (69.9)	1645 (46.6)	
Limitation	702 (59.0)	353 (24.8)	174 (13.0)	126 (7.4)	1355 (22.4)	
Missing or unknown	4 (0.4)	3 (0.2)	0	0	7 (0.1)	
General health						
Excellent	44 (4.0)	99 (8.4)	116 (11.6)	158 (14.7)	417 (10.4)	<.001
Very good	204 (19.5)	297 (28.8)	343 (35.0)	380 (36.4)	1224 (31.2)	
Good	414 (36.0)	469 (37.0)	401 (34.5)	427 (32.5)	1711 (34.8)	
Fair or poor	440 (35.3)	317 (20.6)	216 (13.3)	244 (11.4)	1217 (18.5)	
Missing or unknown	62 (5.2)	67 (5.1)	65 (5.6)	77 (5.0)	271 (5.2)	
Frail or unable to walk^h^						
Yes	444 (36.5)	160 (10.9)	73 (4.9)	55 (2.8)	732 (11.4)	<.001
No	720 (63.5)	1089 (89.1)	1068 (95.1)	1231 (97.2)	4108 (88.6)	

Abbreviations: BMI, body mass index (calculated as weight in kilograms divided by height in meters squared); MVPA, moderate-to-vigorous physical activity intensity; NHANES, National Health and Nutrition Examination Survey.

^a^ Data are presented as the number (%) of US adults unless noted otherwise. Percentages and means (95% CIs) were estimated using US population and study design weights. Sample weights included readjustments after stratification from loss of observations owing to missing accelerometry data, and all participants were eligible for mortality linkage through the National Death Index.

^b^ Total number of minutes per day recorded by the accelerometer that were at or above the cutpoint of 760 counts per minute^4^ (ie, MVPA).

^c^ *P* values were computed using linear regression for continuous variables and logistic regression for categorical variables, and they did not include unknown or missing categories. *P* values were computed separately for each covariate and indicate statistically significant differences between physical activity groups if *P* < .05.

^d^ Healthy Eating Index 2005 scores describe an individual diet quality as recommended by the 2005 Dietary Guidelines for Americans. Scores range from 0 (least healthy) to 100 (most healthy).

^e^ Race and ethnicity was determined by self-report and was classified using preferred terminology from the National Center for Health Statistics as Mexican American, non-Hispanic Black, and non-Hispanic White. Mexican American individuals were oversampled rather than broader groups of individuals from Latin America. The “other” classification includes Alaska Native, Asian, other Hispanic, or other race and ethnicity, including multiracial.

^f^ BMI ranges are as follows: normal weight, 13.4 to 24.9 (includes underweight participants with a BMI <18.5); overweight, 25.0 to 29.9; and obesity, 30.0 to 62.5.

^g^ Mobility limitations were assessed among those aged 60 years or older or among younger individuals reporting some type of physical or mental limitation. A mobility limitation was defined as a report of having difficulty walking for a quarter mile, without special equipment, or walking up 10 steps.

^h^ Frailty was defined as a frail index score greater than 45% derived from items related to limitations of activities of daily living, vision, hearing, health care use, medications, and several physiological and biochemical measurements.^6^ Inability to walk was defined as needing special equipment to walk.

Adjusted hazard ratios changed from 0.69 to 0.28 across increasing activity categories (vs 0-19 minutes per day). Hazard ratios used to generate the PAFs for the 8 activity categories were as follows: 1.00 (reference) for 0 to 19 (548 [7.9%]), 0.69 (95% CI, 0.55-0.85) for 20 to 39 (616 [10.0%]), 0.51 (95% CI, 0.42-0.63) for 40 to 59 (635 [11.8%]), 0.40 (95% CI, 0.29-0.55) for 60 to 79 (614 [12.7%]), 0.34 (95% CI, 0.25-0.47) for 80-99 (633 [14.4%]), 0.32 (95% CI, 0.21-0.48) for 100 to 119 (508 [12.1%]), 0.30 (95% CI, 0.19-0.48) for 120-139 (384 [9.3%]), and 0.28 (95% CI, 0.18-0.42) for 140 or more (902 [21.7%]) minutes per day. The number of participants with frailty or needing special equipment was 280 (49.4%) for 0 to 19, 164 (26.3%) for 20 to 39, 94 (12.4%) for 40 to 59, 66 (9.5%) for 60 to 79, 42 (5.1%) for 80 to 99, 31 (4.7%) for 100 to 119, 20 (2.9%) for 120 to 139, and 35 (2.7%) for 140 or more minutes per day.

Increasing MVPA by 10, 20, or 30 minutes per day was associated with a 6.9%, 13.0%, and 16.9% decrease in the number of deaths per year, respectively. Adding 10 minutes per day of physical activity resulted in an estimated 111 174 preventable deaths per year (95% CI, 79 594-142 754), with greater benefits associated with the addition of more physical activity (209 459 preventable deaths [95% CI, 146 299-272 619] for 20 minutes and 272 297 preventable deaths [95% CI, 177 557-367 037] for 30 minutes) (Figure).

**Figure.**
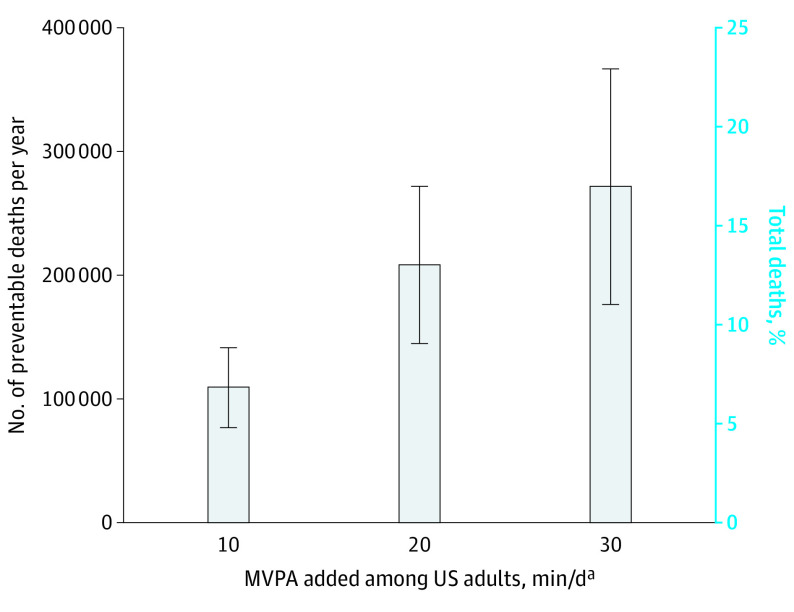
Number of Preventable Deaths and Equivalent Proportion of Total Deaths by Added Amount of MVPA Among US Adults Aged 40 to 85 Years or Older, 2003 to 2006 National Health and Nutrition Examination Survey Hazard ratios were estimated using Cox proportional hazard regression models and the proportional hazards assumption was confirmed for moderate-to-vigorous physical activity intensity (MVPA). Hazard ratios (95% CIs) were used to generate the population attributable fractions (PAFs). When calculating the PAFs, physical activity levels for participants identified as having frailty or needing special equipment to walk were held constant. Bars represent 95% CIs for both the estimated number of deaths and the proportion of total deaths. Hazard ratios and the estimated number of deaths per year were adjusted for age, sex, race and ethnicity, education level, body mass index, diet quality, alcohol consumption, smoking status, self-reported diabetes, heart disease, heart failure, stroke, cancer, chronic bronchitis, emphysema, mobility limitations, and general health. The number of deaths per year was computed using the 2003 annual mortality for US adults aged 40 to 84 years. Models included US population and study design weights to account for the complex survey. Sample weights also included poststratification adjustments from loss of observations attributable to missing accelerometry data, and all participants were eligible for mortality linkage through the National Death Index. ^a^Total number of minutes per day recorded by the accelerometer that were at or above the cutpoint of 760 counts per minute^4^ (ie, MVPA).

The PAFs indicate that the addition of 10 minutes per day of MVPA was associated with the prevention of 8.0% (95% CI, 6.0-10.0) of total deaths per year among men, 5.9% (95% CI, 2.0-9.8) among women, 4.8% (95% CI, 0.0-10.7) among Mexican American individuals, 6.1% (95% CI, 2.2-10.0) among non-Hispanic Black individuals, and 7.3% (95% CI, 5.3-9.3) among non-Hispanic White individuals.

## Discussion

In this cohort study, we estimated that approximately 110 000 deaths per year could be prevented if US adults aged 40 to 85 years or older increased their MVPA by a small amount (ie, 10 minutes per day). Similar benefits were observed for men and women and for Mexican American, non-Hispanic Black, and non-Hispanic White adults. To our knowledge, this is the first study to estimate the number of preventable deaths through physical activity using accelerometer-based measurements among US adults while recognizing that increasing activity may not be possible for everyone. However, 1 week of monitoring may not reflect changes in activity over time, and the observational study design limits the direct determination of causality.

These findings support implementing evidence-based strategies to improve physical activity for adults and potentially reduce deaths in the US.

## Notes

Supplement.
eMethods
