# Supplementary material for: Estimated Number of Deaths Prevented Through Increased Physical Activity Among US Adults
Source: JAMA Intern Med. 2022 Jan 24;182(3):349–52. doi: 10.1001/jamainternmed.2021.7755 (PMC8787676; doi:10.1001/jamainternmed.2021.7755)
Supplement: Supplement. — eMethods [file jamainternmed-e217755-s001.pdf]

# Supplementary Online Content

Saint-Maurice PF, Graubard BI, Troiano RP, et al. Estimated number of deaths prevented through increased physical activity among US adults. *JAMA Intern Med*. Published online January 24, 2022. doi:10.1001/jamainternmed.2021.7755

**eMethods**

This supplementary material has been provided by the authors to give readers additional information about their work.

© 2022 Saint-Maurice PF et al. *JAMA Internal Medicine*.

# eMethods

## Accelerometer-based assessment of physical activity

Accelerometry counts generated from the devices were classified as moderate-to-vigorous physical activity by using an established cut-point of 760 counts per minute.[1,2] This cut-point was calibrated to differentiate light intensity activities from a broader range of moderate-to-vigorous intensity activities, including both activities of everyday living as for example, household chores, gardening, and slow walking, and activities that require walking/ambulation including structured exercise routines and sports.[1,2] This method has been evaluated for validity[2-5] and we have used it in previous activity-mortality studies in the National Health and Nutrition Examination Survey (NHANES), finding a strong inverse non-linear association.[6] In this study we summed all recorded minutes/day that were above this threshold to determine the amount of moderate-to-vigorous intensity physical activity given the consensus that all minutes of activity are important for health.[7]

Of 6,355 adults 40+ years of age invited to wear the monitor, 4,840 agreed and recorded at least one day of valid accelerometry data and were included in our study (Figure). Participants with valid accelerometry data have higher prevalence of obesity (BMI ≥30.0), and current alcohol consumption, but a lower prevalence of heart disease, heart failure, mobility limitations, stroke, and poor health, when compared to remaining participants who did not wear the device or had invalid data (n=1,515).[8] As an adjustment for loss of observations from non-response due to missing accelerometry data we re-post-stratified NHANES sample weights by cross-classification categories of age, sex and race and ethnicity categories to match to US population totals in these categories at the time of the survey. Results were essentially unchanged when compared to not re-post-stratifying the sample weights.

## Calculation of Population Attributable Fraction (PAF) and annual deaths

We first categorized measured moderate-to-vigorous physical activity into 8 exclusive groups, ranging from low (0-19 min/d) to high activity (140+ min/d). Activity groups were defined to characterize range and prevalence of activity levels in the population and the known non-linear relation between physical activity and mortality.[6] We calculated relative risks (hazard ratios) across the eight activity groups (0-19 min/d, reference) adjusting for demographic factors and covariates including age (years), sex, race and ethnicity (non-Hispanic White, non-Hispanic Black, Mexican American, and other), education level (< high school, high school diploma, > than high school), measured body mass index (BMI; <25, 25-29.9, ≥30 kg/m$^2$), diet quality (2005 Healthy Eating Index; continuous), alcohol consumption (never, former, current, unknown), smoking status (never, former, current), self-reported diabetes (yes, no, borderline), heart disease (yes, no, missing/unknown), heart failure (yes, no, missing/unknown), stroke (yes, no), cancer (yes, no), chronic bronchitis (never, former, current, missing/unknown), emphysema (yes, no, missing/unknown), mobility limitation (no limitations, limitations unrelated to mobility, mobility limitations, missing/unknown), and general health (excellent, very good, good, fair, poor, missing/unknown). Relative risks were computed using Cox proportional hazard models with follow-up time defined from baseline through date of death or censoring (12/31/2015), whichever came first.

© 2022 Saint-Maurice PF et al. *JAMA Internal Medicine*.

To calculate the PAFs, we set counterfactuals for increased moderate-to-vigorous physical activity by adding 10, 20, and 30 minutes/day to the observed amount of activity for each participant. The mortality rate is $I\sum r_i p_i$, where I is the population baseline mortality rate, $r_i$ and $p_i$ are the relative risks and prevalence, respectively, corresponding to each combination, i, of activity group and other risk-factors and covariates. and the sum is over risk-factor/covariate combinations. We calculated $r_i^*$ as the "counterfactual" relative risk in which the activity level is set to the increased amount, among individuals who were not frail and didn't need special equipment to walk, while keeping all other factors for each participant unchanged. The number of deaths/year that could be prevented with increased physical activity were calculated as the adjusted PAF[9] x US population annual number of deaths for 2003 among adults aged 40-84 years (n=1,611,227 deaths).[10]

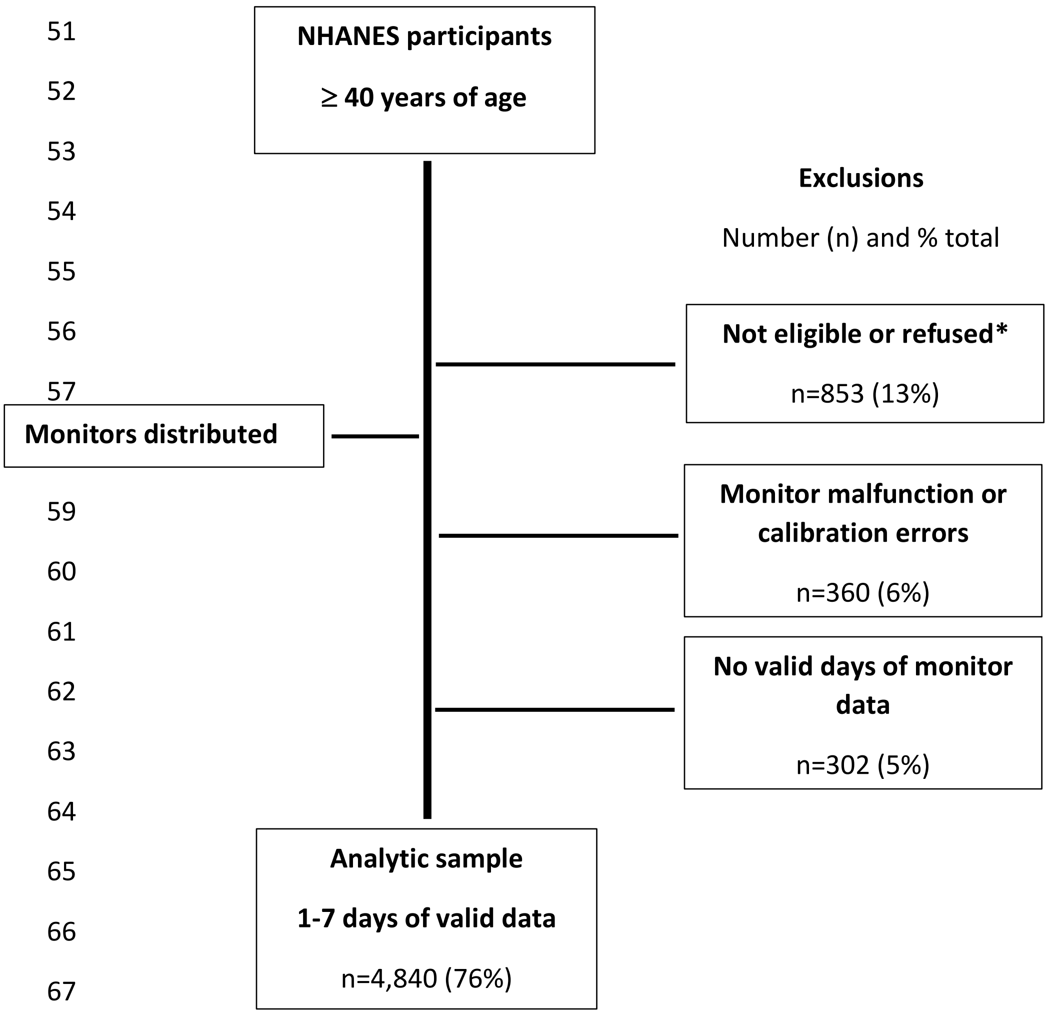


* Participants who were in wheelchairs or who had impairments that precluded them from wearing the monitors were not eligible. NHANES documentation does distinguishes non-eligibility from refusals.

**Figure. Flowchart for analytical sample with monitor data.**

© 2022 Saint-Maurice PF et al. *JAMA Internal Medicine*.

## References


1. Matthews CE. Calibration of accelerometer output for adults. *Med Sci Sports Exerc.* 2005;37(11 Suppl):S512-522.
2. Matthews CE, Keadle SK, Berrigan D, et al. Influence of Accelerometer Calibration Approach on Moderate-Vigorous Physical Activity Estimates for Adults. *Med Sci Sports Exerc.* 2018;50(11):2285-2291.
3. Crouter SE, DellaValle DM, Haas JD, Frongillo EA, Bassett DR. Validity of ActiGraph 2-regression model, Matthews cut-points, and NHANES cut-points for assessing free-living physical activity. *J Phys Act Health.* 2013;10(4):504-514.
4. Matthews CE, Kozey Keadle S, Moore SC, et al. Measurement of Active and Sedentary Behavior in Context of Large Epidemiologic Studies. *Med Sci Sports Exerc.* 2018;50(2):266-276.
5. Welk GJ, McClain JJ, Eisenmann JC, Wickel EE. Field validation of the MTI Actigraph and BodyMedia armband monitor using the IDEEA monitor. *Obesity (Silver Spring, Md).* 2007;15(4):918-928.
6. Matthews CE, Keadle SK, Troiano RP, et al. Accelerometer-measured dose-response for physical activity, sedentary time, and mortality in US adults. *Am J Clin Nutr.* 2016;104(5):1424-1432.
7. Piercy KL, Troiano RP, Ballard RM, et al. The Physical Activity Guidelines for Americans. *JAMA.* 2018;320(19):2020-2028.
8. Saint-Maurice PF, Troiano RP, Bassett DR, Jr., et al. Association of Daily Step Count and Step Intensity With Mortality Among US Adults. *JAMA.* 2020;323(12):1151-1160.
9. Graubard BI, Flegal KM, Williamson DF, Gail MH. Estimation of attributable number of deaths and standard errors from simple and complex sampled cohorts. *Stat Med.* 2007;26(13):2639-2649.
10. Hoyert DL, Heron MP, Murphy SL, Kung HC. Deaths: final data for 2003. *Natl Vital Stat Rep.* 2006;54(13):1-120.


© 2022 Saint-Maurice PF et al. *JAMA Internal Medicine*.
